# Autotherapy

**Published:** 1916-08

**Authors:** 


### Autotherapy.

Since the attention of the medical profession is being turned to autotherapy—and every subject that gives promise of extending professional knowledge should be closely studied—the TEXAS MEDICAL JOURNAL has begun with this issue the publication of a series of short articles from Dr. Duncan, of New York, who is an authority on that subject. Autotherapy may or may not be all that is claimed for it—most new discoveries claim too much—but it certainly behooves every progressive physician to study its claims and come to some definite conclusion as to its merits. There is something about some of the suggestions in the articles that are a bit repugnant to refined tastes, but where life is at stake refined tastes should be relegated to the saving of life. Certainly the learned writer has given much thought to the subject, and he offers evidence of the efficiency of the methods in use, such as no physician can afford to ignore, and therefore all will read what he has to say with a view to getting whatever good there may be in it, rather than to criticize the discovery because of its newness. Whatever progress has been made in medicine has been made in the face of the severest criticism and ridicule, the profession being loath to adopt anythiny new or to concede that those of this day can know much more about the science of medicine than was known by the forefathers. However, the medical mind has been so often forced to accept new theories and treatments in the past few decades that things new and even radical are no longer startling as they once were.

